# Accuracy of automatic couch corrections with on‐line volumetric imaging^*^

**DOI:** 10.1120/jacmp.v10i4.3056

**Published:** 2009-10-07

**Authors:** Winnie Li, Douglas J. Moseley, Tony Manfredi, David A. Jaffray

**Affiliations:** ^1^ Radiation Medicine Program Princess Margaret Hospital Toronto Ontario Canada; ^2^ Department of Radiation Oncology University of Toronto Toronto Ontario Canada; ^3^ Department of Medical Biophysics University of Toronto Toronto Ontario Canada; ^4^ Elekta Inc Crawley UK

**Keywords:** cone‐beam CT, image guidance, automated couch movement, residual error

## Abstract

The purpose of this study was to characterize automatic remote couch adjustment and to assess the accuracy of automatic couch corrections following localization with cone‐beam CT (CBCT). Automatic couch movement was evaluated through passive reflector markers placed on a phantom, tracked with an optical tracking system (OTS). Repeated couch movements in the lateral, cranial/caudal, and vertical directions were monitored through the OTS to assess velocity and response time. In conjunction with CBCT, remote table movement for patient displacements following initial setup was available on four linear accelerators (Elekta Synergy). After the initial CBCT scan assessment, patients with isocenter displacements that exceeded clinical protocol tolerances were corrected using remote automatic couch movement. A verification CBCT scan was acquired after any remote movements. These verification CBCT datasets were assessed for the following time periods: one month post clinical installation, and six months later to monitor remote couch correction stability. Residual error analysis was evaluated using the verification scans. The mean ± standard deviations (μ±σ) of couch movement based on phantom measurements with the OTS were 0.16±0.48mm,0.32±0.30mm,0.11±0.12mm in the L/R, A/P, and S/I couch directions, respectively. The fastest maximum velocity was observed in the inferior direction at 10.5 mm/s, and the slowest maximum velocity in the left direction at 3.6 mm/s. From 1134 verification CBCT registrations for 207 patients, the residual error for each translational direction from each month evaluated are reported. The μ was less than 0.3 mm in all directions, and σ was in the order of 1 mm. At a 3 mm threshold, 21 of the 1134 fractions (2%) exceeded tolerance, attributed to patient intrafraction movement. Remote automatic couch movement is reliable and effective for adjusting patient position with a precision of approximately 1 mm. Patient residual error observed in this study demonstrates that displacement is minimal after remote couch adjustment.

PACS number: 87.55.Qr, 87.56.bd, 87.57.Q

## I. INTRODUCTION

The goal of radiation therapy is to eradicate tumor cells while minimizing dose to surrounding normal tissue. Recent advances in image‐guided radiation therapy (IGRT) processes have increased the precision and reproducibility of radiotherapy treatments. To assess patient positioning and monitor potential changes throughout treatment, three‐dimensional (3D) kilovoltage (kV) imaging provides enhanced patient setup information over traditional two‐dimensional (2D) orthogonal megavoltage (MV) portals.^(^ ^1^ ^–^ ^6^ ^)^ Volumetric imaging has a positive impact on patient positioning as front‐end users are able to visualize anatomy on a 3D scale, with the ability to monitor patient displacements and soft tissue changes.^(5)^

Since clinical implementation of volumetric imaging at our institute in December 2005, it has served as a “3D portal imager” for daily IGRT. Through an on‐line volumetric imaging process, setup errors are detected by registration of the cone‐beam computed tomography (CBCT) to the reference planning CT data set.^(4)^ With its application on a variety of anatomical sites, volumetric imaging through the X‐ray Volumetric Imaging (XVI) software (v3.5, Elekta, Stockholm, Sweden) became rapidly adopted in the clinical environment,^(^ ^2^ ^,^ ^3^ ^,^ ^7^ ^)^ increasing the geometric precision and accuracy of therapy by reducing systematic and random setup errors.^(^ ^6^ ^,^ ^8^ ^)^ Radiotherapy treatments are image‐guided using the 3D images and set action thresholds. If the scan displacement exceeds threshold, an intervention in the form of a manual couch adjustment from inside the treatment room was performed, and a second (verification) CBCT followed to assess residual error. The reported accuracy of residual error after couch corrections with an unambiguous rigid phantom is within a few hundreds of a millimeter.^(8)^

At our institution, the remote couch functionality became available for clinical use in conjunction with patient imaging in October 2006. With the implementation of remote couch table movement, the offset of the planning CT to the CBCT, as measured by the imaging software, can be carried out automatically from outside the treatment room. Without having to reenter the treatment room to adjust the treatment couch, clinical workflow is improved. A humanoid phantom in conjunction with a Polaris optical tracking system (Northern Digital Inc., Canada) was used during the installation of the first remote couch to assess the stability and accuracy of couch movements. Subsequent testing and commissioning procedures were done on four linear accelerators (Elekta Synergy, Stockholm, Sweden) prior to clinical implementation.

This paper describes the initial experience of remote couch commissioning and clinical implementation at our institute, and reports: a) an evaluation of the dynamics of couch movement and control through an optical tracking system, b) phantom‐based studies of the residual error accuracy and stability of couch corrections across four linear accelerators, and c) the residual error for patients following automatic couch adjustments for two time periods ‐ one month following installation, and six months later to assess stability.

## II. MATERIALS AND METHODS

### A. Performance of couch movement measured by an optical tracking system

**Figure 1:**
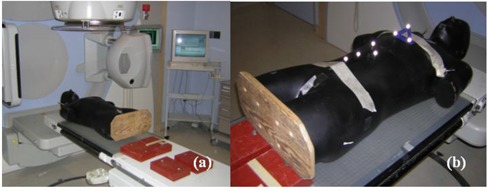
Setup on linac for couch positioning accuracy: (a) a humanoid phantom (90 kg in total) placed in the headfirst supine treatment position; (b) two optical localization tools taped on chest.

On the first remote automated couch installed in the clinic, the reliability of couch movement was tested with an optical tracking system. The couch was loaded with the humanoid phantom^(9)^ and four steel counter‐weight bricks to simulate a patient mass of 90 kg (Fig. 1(a)). Passive stereoscopic devices (optical reflective spheres 1 cm in diameter) were attached to the phantom's torso to enable optical tracking (Fig 1(b)). The optical tracking system consists of custom software on a personal computer connected to the Polaris infrared stereoscopic camera (Northern Digital Inc., Waterloo, Canada).^(10)^ The camera was initially calibrated to the linear accelerator's isocenter, and then transmitted three‐dimensional position and orientation information by tracking the spherical infrared reflective markers placed on the phantom's torso. The sampling rate was approximately 18 Hz.

In the head first supine position, the phantom was displaced from the isocenter position on the table through remote automatic table movements. Repeated motions of ±10mm in each translational direction, as determined by the couch digital readout, were performed to simulate a threshold limit of allowable patient remote movement in the clinical setting. The cycle was repeated seven times in the left‐right (L/R) direction (couch lateral), ten times in the anterior‐posterior (A/P) direction (couch vertical), and twelve times in the superior‐inferior (S/I) direction (couch longitudinal). This yielded 14 left and 14 right measurements, 20 anterior and 20 posterior measurements, and 24 superior and 24 inferior measurements. The couch location with respect to isocenter and bed velocity was measured through optical tracking. Couch movements were initiated remotely from the console using the linac control system. The time to reach the new position, average couch velocity, and final positions of the translational directions were recorded.

### B. Remote couch reliability: commissioning results

Four linacs were upgraded with the remote couch functionality in succession. During the commissioning process on each linac, the phantom was set up in four orientations: headfirst supine (HFS), headfirst prone (HFP), feetfirst supine (FFS), and feetfirst prone (FFP). For each orientation, the thoracic vertebral bodies were targeted, defined through a region of interest called the “clipbox”. For each orientation, arbitrary displacements of magnitude no greater than 2 cm in any translational axes were randomly created, and a CBCT followed to measure the displacement. Image registration used a histogram based gray‐value matching technique based on the standardized region of interest.^(11)^ After detection of displacements through the volumetric image matching software, translational couch shifts were communicated to the linac control system and remotely activated through the function keypad at the treatment console area. A second CBCT followed each couch movement to ensure table accuracy, direction of table movement, and to quantify couch residual error. These measurements were performed within a single session (1 hour) on each unit, and resulted in 16 residual error measurements for the phantom.

### C. Clinical investigation: patient residual error

#### C.1 Patients

Patient data was collected in two periods for analysis, approved by an Institutional Research Ethics Board. The first remote couch table was clinically available in October 2006, and the fourth one in November 2006. For all patients at time of treatment, a CBCT image set was acquired after initial alignment, and compared to the planning reference CT through the volumetric imaging software. If all translational parameters were within 3 mm, patient treatment would commence. If any translational parameter exceeded 3 mm, the treatment couch was adjusted, and a second (verification) CBCT was acquired to assess residual setup error prior to treatment. All patients with a second/verification CBCT scan done on each of the four machines’ imaging database one month following general installation were analyzed retrospectively. This group constituted the first time period, and included a total of 107 patients. Six months later, the data collection was repeated to assess remote couch movement stability, and included 100 patients.

#### C.2 Image‐guidance methodology

Patients were treated on a linear accelerator (Elekta Synergy, Stockholm, Sweden), with an orthogonal mounted kV source and amorphous silicon fat panel detector.^(12)^ The volumetric CBCT scans were acquired over 2 minutes with a gantry rotation of 360°, a frame rate of 5.5 Hz, and reconstructed with approximately 650 projections, reconstructed at 400×400×256 voxels with a 1 mm isotropic voxel size. Depending on the site of interest, the field of view (FOV), kVp, mA, and ms were adjusted for dose and image quality. The imaging technique and dose per volumetric scan at a 2 cm depth for each anatomical site ranged as follows: 120kVp, 20mA, 40ms, and 13mGy for head and neck; 120kVp, 20mA, 20ms, and 6 mGy for thorax; 120kVp, 20mA, 40ms, and 13 mGy for pelvis; and 120kVp, 32mA, 20ms, and 10mGy for lower limb sarcoma.^(13)^

#### C.3 Data collection

In the retrospective analysis, any verification images for each patient during the two months of interest were automatically reregistered off‐line with a standardized region of interest (clipbox), focused around bony anatomy. The clipbox used was the same as that used in clinical practice. For all patients reported, the setup error was calculated by registration of the planning CT to the CBCT scan using bony anatomy as a surrogate for tumor position. Registration of the CBCT datasets to the reference CT was done using automatic algorithms without manual adjustments to eliminate interobserver variability and subjectivity.^(14)^ A visual inspection of the images was done after automatic registration in the coronal, sagittal, and transverse planes, and displacements in all six degrees of freedom were recorded. The displacement values were then noted for the L/R, S/I, and A/P translational planes, as well as the L/R (pitch), S/I (roll), and A/P (yaw) rotational planes. This analysis resulted in a total of 1134 3D registrations for 207 patients performed by a single observer.

#### C.4 Analysis

The mean (μ) and standard deviation (σ) were calculated for the results from the first month of clinical service, and six months later. Statistical comparison of the two months was performed using a Student's t‐test. The μ, σ, and 95% confidence interval were also calculated for each anatomic site.

## III. RESULTS

### A. Evaluation of couch movements through optical tracking

**Figure 2:**
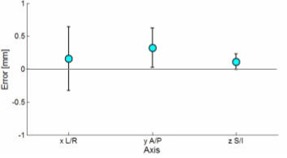
Final positioning error: remote motions of ±10mm were repeated 10 times in each of the cardinal directions; mean error was 0.16, 0.32, 0.11 mm in the L/R, A/P, S/I directions; standard deviation for measurements was 0.48, 0.30, 0.12 mm.

**Figure 3:**
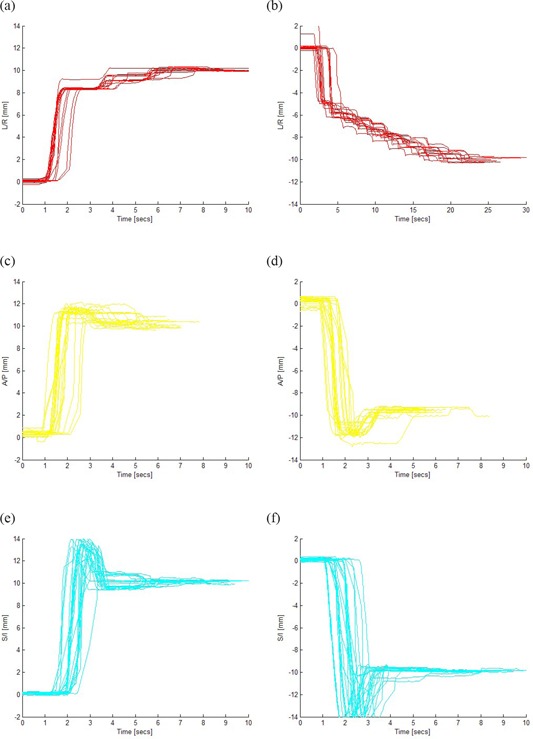
Optical tracking traces for phantom position: sampling rate approximately 18 Hz; traces shown for 10 mm couch moves in the following directions: a) right, b) left, c) anterior, d) posterior, e) inferior, f) superior. The slowest response time was in the left lateral direction with 25 seconds (note the difference in horizontal scale); the fastest in the superior/inferior directions at 6 seconds.

The results of couch movement are seen in Fig. 2. The mean error was 0.16 mm, 0.32 mm, 0.11 mm in the L/R, A/P, and S/I directions, with a standard deviation of 0.48 mm, 0.30 mm, and 0.12 mm, respectively. The response time of the couch to seek the desired correction (10 mm) differed, depending on translational axis and direction. Optical traces for the measurements are seen in Fig. 3. The average time and maximum velocity to reach a displacement of 10 mm for all translational directions are shown in Table 1. For the remote couch characterized, the lateral movements had the slowest movements and settling rate. There was also asymmetry in movement as the left lateral direction had the largest average couch‐settling rate with the slowest velocity at 3.1 mm/second. The fastest couch movement was observed in the cranial/caudal axis. In the superior direction (longitudinal couch movement), the average time for the couch to reach the desired position was 6 seconds, while in the inferior direction the average setting time was 5 seconds.

**Table 1:** Characterization of remote couch movement through optical tracking. The average response time (s) and maximum couch velocity (mm/s) to reach a displacement of 10 mm for all translational directions are shown.

	*Average Time(s)*	*Maximum Velocity (mm/s)*
Left	25.0	3.1
Right	8.0	5.6
Anterior	5.5	6.7
Posterior	5.5	7.7
Superior	6.0	10.0
Inferior	5.0	10.5

### B. Commissioning results for four linacs

The residual error in repositioning of the phantom, scanned in four orientations during couch commissioning on four linear accelerators, is seen in Table 2. The mean and standard deviations were calculated for each phantom orientation. The μ was less than 0.4 mm in any direction and orientation, while o ranged between 0.1–0.6 mm. The phantom measurements were reproducible, and the results were independent of phantom orientation and treatment unit.

**Table 2:** Final positioning error for phantom measurements following CBCT imaging and remote auto couch adjustments. The phantom was scanned in four orientations, and the mean (μ) and standard deviation (σ) were calculated.

	*Headfirst Supine*			*Feetfirst Supine*			*Feetfirst Prone*			*Headfirst Prone*		
	*X (mm)*	*Y (mm)*	*Z (mm)*	*X (mm)*	*Y (mm)*	*Z (mm)*	*X (mm)*	*Y (mm)*	*Z (mm)*	*X (mm)*	*Y (mm)*	*Z (mm)*
Linac 1	0.0	0.2	0.0	−0.3	0.6	0.0	−0.1	0.3	−0.7	−0.6	0.1	1.0
Linac 2	−0.7	0.8	−0.2	0.2	0.3	−0.2	−0.8	−0.5	0.0	−0.1	−0.2	−0.4
Linac 3	0.3	−0.7	−0.3	−0.2	0.2	0.1	0.7	0.1	−0.4	0.5	−0.2	−0.3
Linac 4	−0.1	−0.3	0.0	−0.3	0.3	0.1	0.0	0.4	−0.5	−0.1	0.7	0.2
μ	−0.1	**0.0**	−0.1	−0.2	**0.4**	**0.0**	−0.1	**0.1**	−0.4	−0.1	**0.1**	**0.1**
σ	**0.4**	**0.6**	**0.2**	**0. 2**	**0.2**	**0.1**	**0.6**	**0.4**	**0.3**	**0.5**	**0.4**	**0.6**

### C. Patient residual error

**Figure 4:**
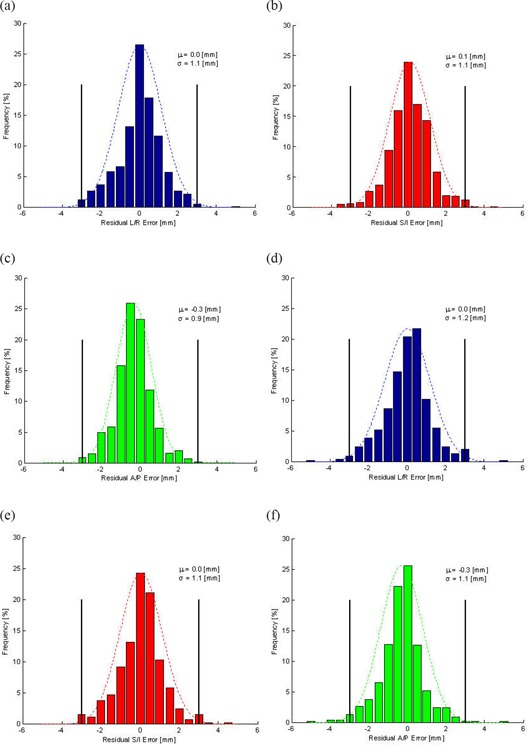
Patient residual error. Results for the first month of clinical service for four linacs are seen in a), b) and c) for the left‐right (L/R), superior‐inferior (S/I), and anterior‐posterior (A/P) directions, respectively. Results for the first month of clinical service following remote couch QA for four linacs are seen in d), e) and f) for the left‐right (L/R), superior‐inferior (S/I), and anterior‐posterior (A/P) directions, respectively. The black dotted lines represent the ±3mm threshold. The mean (μ) and standard deviation (σ) are noted for each direction.

Translational (L/R, A/P, S/I) patient residual error for the two months of couch verification data collection is shown in Fig. 4. The first month of clinical patient data following remote couch installation are seen in Fig. 4(a) to 4(c). The second month of residual error analysis followed six months later, and results are seen in Fig. 4(d) to 4(f). In the L/R direction, residual error (μ±σ) was 0.0±1.1mm and 0.0±1.2mm for the two months, respectively. In the S/I direction, residual error (μ±σ) was ‐ 0.3±0.9mm and ‐ 0.3±1.1mm for the two months, respectively. Finally, in the A/P direction, residual error (μ±σ) was 0.1±1.1mm and 0.0±1.1mm for the two months, respectively. The results of the t‐test between the two months were p=0.8911(L/R),p=0.5663(S/I), and p=0.3038(A/P). As there was no clinical significance between the two time periods, this suggests there is stability in the volumetric imaging/registration system and corresponding automatic couch movements.

Table 3 presents both months’ data, separated by anatomic site. At a 3 mm threshold, none of the head and neck patients (n=32) exceeded tolerance. For the thoracic/abdomen cohort (n=150), 1.24% of the measured fractions (11 of 884) exceeded 3 mm. In the pelvis patients (n=12), 3.28% of the measured fractions (2 of 61) exceeded tolerance, and for the sarcoma cohort (n=13), 8.51% of the measured fractions (8 of 94) exceeded threshold. The largest percentage of the patients who exceeded tolerance was lower limb sarcoma patients, where the effects of rotation predominate, and the limited field of view may hinder the registration volume (i.e. absence of joints in a long bone). As the remote couch only corrects translational (L/R, S/I, A/P) displacements, rotational errors are still present in verification CBCTs. These rotations may alter slightly through patient intrafraction motion, changing the translational displacements calculated for residual error analysis. The other outliers in the population consist of patients with intrafraction motion, either from patient movement due to discomfort or extreme respiratory motion (i.e. coughing). Clinically, these patients would have been reshifted with the remote couch prior to radiation beam delivery. A third CBCT would not have been acquired due to time and workflow constraints.

**Table 3:** Results by anatomical region, where N represents the number of patients. Translational and rotational residual errors are noted with the mean (μ), standard deviation (σ), and 95% confidence interval (CI). A total of 207 patients and 1134 verification CBCT registrations are represented.

		*R‐L (mm)*	*S‐I (mm)*	*A‐P (mm)*	*Pitch (°)*	*Roll (°)*	*Yaw (°)*
**Head & Neck** N=32	μ	0.1	−0.1	−0.1	0.3	−0.3	−0.3
**95 fractions**	σ	0.9	1.0	1.1	1.4	1.7	1.3
	**95% CI**	1.7	1.9	2.1	2.7	3.2	2.4
**Thorax/ Abdo** N=150	μ	0.0	0.1	−0.3	1.1	−0.9	0.1
884 fractions	σ	1.2	1.0	1.0	1.5	1.6	1.6
	**95% CI**	2.3	2.0	1.9	2.9	3.1	3.1
**Pelvis** N=12	μ	−0.2	0.0	−0.4	0.7	−0.2	−0.2
**61 fractions**	σ	1.0	0.8	1.2	1.5	1.2	0.8
	**95% CI**	1.9	1.6	2.4	2.9	2.3	1.5
**Lower Limb Sarcoma**	μ	0.0	0.3	−0.6	−0.1	−0.3	−0.5
N=1394fractions	σ	1.3	1.7	1.0	1.6	2.1	1.0
	**95% CI**	2.5	3.2	2.0	3.2	4.0	1.9

## IV. DISCUSSION

The acceleration, final speed, and deceleration of remote couch movement are adjusted using service software configurable items. These items are calculated to deliver the required operational parameters, and compensate for variability in mechanical and electrical sub‐systems. The software items cannot compensate for extremes of mechanical stiffness or slippage in the clutches and gearing of the table. A decrease in velocity in the lateral couch directions was noted with the couch assessed with the OTS, especially in the left lateral direction with an average time of 25 seconds to travel 10 mm. The cause was determined to be a defective clutch in the lateral drive assembly, which was subsequently replaced. In all translational directions, the acceleration and velocity of couch movements were acceptable for clinical use.

The maximum remote movement allowed for the automatic couch is 2.0 cm from the treatment console. Any parameter greater than 2.0 cm would be adjusted inside the treatment room using the hand pendant. However, this is not clinically relevant at our institution as the maximum allowed automatic movement is 1.5 cm. If a patient's setup exceeds the image guidance threshold of 1.5 cm, the corrective measure is to reenter the treatment room, and reinitiate patient setup. Therefore, at our institute, the limitation set by the remote automatic couch movement does not affect clinical operations.

Patient residual data was collected at one and six months after remote couch installation, between which novel quality assurance (QA) processes were introduced to the clinic. The initial QA procedure focused upon the calibration and coincidence of the MV/kV isocenter. ^(^ ^8^ ^,^ ^15^ ^)^ With the remote couch system, a novel and revised QA procedure was implemented to ensure daily remote couch movement is accurate and reliable.^(16)^ Briefy, using the QA phantom (Penta‐Guide, QUASAR, London, Canada), lasers inside the treatment room were aligned to a setup point with a known displacement. A CBCT would be acquired at the offset, and the imaging software would calculate the offset of the embedded spheres within the phantom. Automated couch movements would be initiated to achieve the offset to the calculated isocenter position, followed by confirmation MV orthogonal portal images. The QA process validated the accuracy of known couch movements while still evaluating kV/MV isocenter coincidence. Such QA activities are carried out on each linear accelerator daily to ensure stability of the system.

The daily QA procedure is necessary to monitor and detect errors with the remote couch. The QA procedure failed tolerance after remote couch shifts on one linac in April 2007. The MV images, as well as the laser alignment on the QA phantom, were displaced in the lateral direction by over 2 mm after the indicated value with remote couch shift. As the remote couch functions on a “servo” system, potentiometers are used to gauge the movement specified by the imaging software to the couch. Further investigation showed that the potentiometers on the couch had failed, and had to be recalibrated. As the QA test is sensitive enough to detect positional error in all planes, it has to be performed daily to ensure quality of care and accuracy of guidance.

Decreasing treatment times for a patient undergoing radiotherapy is advantageous as it decreases the amount of time one has to be immobilized on the treatment bed. Validation of the remote couch and its calibration has lead to decreased treatment times for patients. As staff no longer have to reenter the treatment room to manipulate the couch to correct patient offset, this creates a timesaving of approximately 30 seconds. Verification CBCTs (approximately 2 minutes for full rotation image acquisition, approximately 1.5 minute for analysis) may be eliminated, provided that the daily QA procedure is carried out to ensure the remote couch is working in a consistent and dependable manner. Elimination of the extra verification scans and subsequent analysis saves approximately 3.5 minutes per case visit. While it is a general estimate, over the clinical day with a workload of 30 patients, this could potentially lead to a time savings of 90 minutes. This extra time is beneficial for patient assessment and other patient care related activities.

Hornick et al.^(17)^ explained the use of a remote table with tilt and roll operations in 1998. More recently, robotic couches (HexaPOD, Medical Intelligence) with six degrees of freedom have been reported to correct displacements in all directions, but may induce secondary motion.^(18)^ The table installed at our clinic only adjusts for translational shifts. Patients are alone inside the treatment room when the remote bed is activated; therefore, for the first few treatments, it is standard practice at our institute to tell patients via the intercom system that a bed adjustment is being made. The patient is monitored continuously as the couch shifts are carried out to identify any gross patient movements. Such precautions are necessary as unexpected couch moves may startle the patient and change their positioning. In addition to bed movement, due to the “servo” nature of the couch there is an audible clicking sound of the bed associated with the brake release mechanism as it adjusts to the final corrected position. While the patients have commented that they sense the couch move, being informed of the procedure and the associated movement reduces any alarm they may experience. Also, the speed and magnitude of movements are small, so patients do not sense the acceleration or notice any abrupt movement.

As seen in Table 3, the residual error present in the patient population may be explained by anatomical region and associated immobilization. The rigid immobilization for the head and neck group is the S‐frame (MedTech, Orange City, IA), which may account for the increased accuracy of initial setup and reproducibility. Thoracic residual errors were most relevant in the rotational planes, partially explained by a lack of reproducibility of setup tattoos in the region due to respiratory motions. The pelvic patients in this study cohort were mostly prone anal canal patients, where increased rotational displacements (pitch) were detected. Lower limb sarcoma patients observed the highest measured rotations, primarily due to tumor location. Long bones are not always completely encompassed by the imaging FOV, and interpretation of displacements, especially in the S/I direction, can be a challenge. Additionally, sarcomas are often associated with tumor swelling, volume changes, and limb edema.^(^ ^19^ ^,^ ^20^ ^)^ Hence, the limb may no longer fit into the custom immobilization device, decreasing stability and reproducibility of setups.

## V. CONCLUSIONS

The remote automatic table movement is accurate and reliable when used in conjunction with the volumetric imaging software, and is stable and safe for clinical use. Residual error, as measured for phantom and patients, is minimal after remote couch adjustment. The stability of remote couch movements can be monitored daily through novel QA processes.

## ACKNOWLEDGEMENTS

The authors would like to thank Kevin Brown at Elekta for his input.
